# Case Report: Cytomegalovirus Reactivation and Pericarditis Following ChAdOx1 nCoV-19 Vaccination Against SARS-CoV-2

**DOI:** 10.3389/fimmu.2021.784145

**Published:** 2022-01-18

**Authors:** Marlene Plüß, Kemal Mese, Johannes T. Kowallick, Andreas Schuster, Désirée Tampe, Björn Tampe

**Affiliations:** ^1^ Department of Nephrology and Rheumatology, University Medical Center Göttingen, Göttingen, Germany; ^2^ Institute of Medical Microbiology and Virology, University Medical Center Göttingen, Göttingen, Germany; ^3^ Institute of Diagnostic and Interventional Radiology, University Medical Center Göttingen, Göttingen, Germany; ^4^ German Center for Cardiovascular Research (DZHK), Partner Site Göttingen, Göttingen, Germany; ^5^ Department of Cardiology and Pneumology, University Medical Center Göttingen, Göttingen, Germany

**Keywords:** SARS-CoV-2 vaccination, ChAdOx1 nCoV-19, cytomegalovirus, viral reactivation, long COVID

## Abstract

As the coronavirus disease 2019 (COVID-19) pandemic is ongoing and new variants of severe acute respiratory syndrome coronavirus type 2 (SARS-CoV-2) are emerging, there is an urgent need for vaccines to protect individuals at high risk for complications and to potentially control disease outbreaks by herd immunity. Surveillance of rare safety issues related to these vaccines is progressing, since more granular data emerge about adverse events of SARS-CoV-2 vaccines during post-marketing surveillance. Varicella zoster virus (VZV), Epstein-Barr virus (EBV) and cytomegalovirus (CMV) reactivation has already been reported in COVID-19 patients. In addition, adverse events after SARS-CoV-2 mRNA vaccination have also been in the context of varicella zoster virus (VZV) reactivation and directly associated with the mRNA vaccine. We present the first case of CMV reactivation and pericarditis in temporal association with SARS-CoV-2 vaccination, particularly adenovirus-based DNA vector vaccine ChAdOx1 nCoV-19 against SARS-CoV-2. After initiation of antiviral therapy with oral valganciclovir, CMV viremia disappeared and clinical symptoms rapidly improved. Since huge vaccination programs are ongoing worldwide, post-marketing surveillance systems must be in place to assess vaccine safety that is important for the detection of any events. In the context of the hundreds of millions of individuals to be vaccinated against SARS-CoV-2, a potential causal association with CMV reactivation may result in a considerable number of cases with potentially severe complications.

## Introduction

As the coronavirus disease 2019 (COVID-19) pandemic is ongoing and new variants of severe acute respiratory syndrome coronavirus type 2 (SARS-CoV-2) are emerging, there is an urgent need for vaccines to protect individuals at high risk for complications and to potentially control disease outbreaks by herd immunity (1, 2). The European Medicines Agency (EMA) approved the use of vaccines containing a nucleoside-modified messenger RNA (mRNA) that encodes the viral spike glycoprotein (S) of SARS-CoV-2, or an adenovirus-based DNA vector vaccine encoding the SARS-CoV-2 S glycoprotein. Surveillance of rare safety issues related to these vaccines is progressing, since more granular data emerge about adverse events of SARS-CoV-2 vaccines during post-marketing surveillance (3). Varicella zoster virus (VZV), Epstein-Barr virus (EBV) and cytomegalovirus (CMV) reactivation has already been reported in COVID-19 patients (4–10). In addition, adverse events after SARS-CoV-2 mRNA vaccination have also been reported in the context of varicella zoster virus (VZV) reactivation and directly associated with the mRNA vaccine (11, 12). To our knowledge, this is the first report of CMV reactivation after SARS-CoV-2 vaccination, particularly adenovirus-based DNA vector vaccine ChAdOx1 nCoV-19 against SARS-CoV-2.

## Case Description

**Figure 1:**
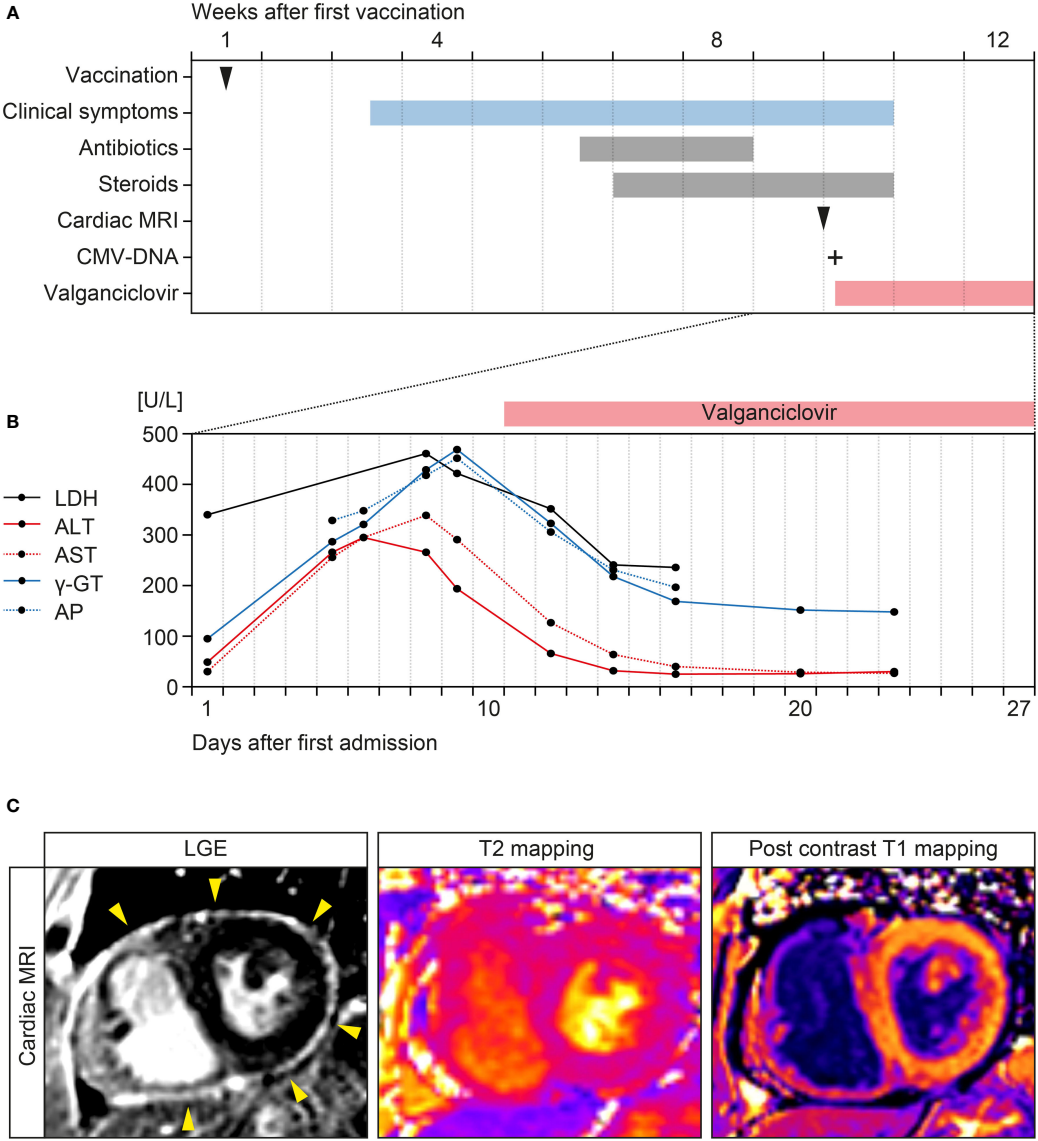
Timeline of the case and cardiac MRI findings. **(A)** Time of ChAdOx1 nCoV-19 vaccination, onset of clinical symptoms, and treatment regimens. **(B)** Time course of laboratory parameters including LDH, AST, ALT, γ-GT, and AP after admission. **(C)** LGE confirming circumferential thickening and contrast enhancement of the entire pericardium (yellow arrowheads) without presenting myocardial enhancement. Advanced tissue characterization with T2 mapping and post contrast T1 mapping did not show evidence of myocardial edema or myocardial enhancement, confirming the diagnosis of isolated pericarditis. ALT, alanine aminotransferase; AP, alkaline phosphatase; AST, aspartate aminotransferase; CMV, cytomegalovirus; γ-GT, γ-glutamyl transferase; LGE, late gadolinium enhancement; MRI, magnetic resonance imaging.

A 67-year-old Caucasian female with a past medical history of atrial fibrillation, hypertension, obesity, degenerative knee joint disease, and no documented history of COVID-19 received a first dose of ChAdOx1 nCoV-19 vaccination. The patient had no allergies, no history of immune deficiency, no recent infectious disease, and denied illicit drug use. Two weeks after vaccination, the patient suffered from fever, weakness and arthralgia of the knees, hips and shoulders (**Figure 1A**). After additional 3 weeks, the patient was admitted to a community hospital with stable vital parameters and normal physical examination (**Figure 1A**). Computed tomography (CT) scans of the chest and abdomen revealed reactive mediastinal lymphadenopathy and hepatic steatosis. Infectious endocarditis was excluded by transesophageal echocardiography (TEE) and repeat blood cultures were all tested negative. Due to elevated IgM titers, IgM gammopathy was suspected but excluded by bone marrow biopsy. Because both, antibiotic treatment with piperacillin/tazobactam and steroids did not improve clinical symptoms with persistent fever and weakness, the patient was referred to our tertiary center three weeks thereafter (**Figure 1A**). A repeat nasopharyngeal swab for SARS-CoV-2 RNA testing by polymerase chain reaction (PCR) was negative. Laboratory examination at admission revealed leukocytosis (16,100/µL, reference range: 4,000-11,000/µL), acute kidney injury (serum creatinine: 1.59 mg/dL, reference range: 0.5-1 mg/dL), hyponatremia (127 mmol/L, reference range: 136-145 mmol/L), elevated C-reactive protein (CRP: 97.9 mg/L, reference range: ≤5 mg/L) and lactate dehydrogenase (LDH: 340 U/L, reference range: 125-250 U/L). During the further disease course, elevated liver transaminase levels including alanine aminotransferase (ALT: 339 U/L, reference range: ≤34 U/L), aspartate aminotransferase (AST: 295 U/L, reference range: ≤31 U/L), canalicular enzymes γ-glutamyl transferase (γ-GT: 469 U/L, reference range: 9-36 U/L), and alkaline phosphatase (AP: 452 U/L, reference range: 40-150 U/L) were detectable (**Figure 1B**). Serological examination excluded hepatitis B virus (HBV), hepatitis C virus (HCV), hepatitis E virus (HEV) infection, and human immunodeficiency virus (HIV). Cardiac magnetic resonance imaging (MRI) confirmed diagnosis of pericarditis with circumferential thickening and contrast enhancement of the entire pericardium at late gadolinium enhancement (LGE, **Figure 1C**) (13, 14). Based on these imaging findings, heart involvement with viral pericarditis was suspected. EBV serology was compatible with past infection (anti-EBV-VCA-IgG: positive, anti-EBV-IgM: negative), confirmed by PCR with no detectable EBV-DNA. However, serology of CMV was compatible with active CMV infection (anti-CMV-IgG: >250 IU/mL, anti-CMV-IgM: positive), confirmed by PCR with detectable CMV viremia (415 IU/mL). Based on active CMV infection, oral valganciclovir was initiated (900 mg twice daily, **Figure 1B**). During the further course, clinical symptoms improved, and liver enzymes normalized (**Figure 1A**). This was associated with successful treatment and decrease of CMV viremia (69.8 IU/mL). The patient was transferred to dermatological care for the treatment of allergic dermatitis after the aforementioned antibiotic treatment, a skin biopsy revealed neither auto-immune nor viral cause of the dermatitis. Thereafter, the patient remained free of symptoms and was discharged with recommendation to continue the antiviral therapy for 3 weeks in total (**Figure 1A**). A follow-up visit 54 days after discharge confirmed normal range liver enzymes (ALT: 9 U/L, reference range: ≤34 U/L; AST: 27 U/L, reference range: ≤31 U/L; γ-GT: 28 U/L, reference range: 9-36 U/L; AP: 70 U/L, reference range: 40-150 U/L) and absence of CMV viremia (<35 IU/mL). In summary, we here present the clinical course of a patient with CMV reactivation and pericarditis in temporal association with ChAdOx1 nCoV-19 vaccination against SARS-CoV-2.

## Discussion

To our knowledge, we present the first case of CMV reactivation and pericarditis following SARS-CoV-2 vaccination, particularly adenovirus-based DNA vector vaccine ChAdOx1 nCoV-19 against SARS-CoV-2. Myocarditis has previously been reported in the context of mRNA vaccines against SARS-CoV-2 (15–21). Myocarditis after SARS-CoV-2 mRNA vaccination developed rapidly in younger patients, mostly after the second vaccination (22). In addition, pericarditis has also been observed predominantly affecting older patients occurring with latency to either the first or second dose of SARS-CoV-2 mRNA vaccine (22). However, a causal connection is still a matter of debate and requires further investigation (23). Although we cannot exclude direct effects of the adenovirus-based DNA vector vaccine contributing to pericarditis, the presence of CMV viremia with typical clinical symptoms and MRI findings, and improvement of symptoms after initiation of antiviral therapy with disappearance of CMV viremia makes CMV reactivation as the primary cause of the observed pericarditis very likely. Moreover, the temporal association between ChAdOx1 nCoV-19 vaccination and CMV viremia thereafter could implicate a causal connection between SARS-CoV-2 vaccination and CMV reactivation. This is especially relevant since pericarditis has previously been observed in the context of active CMV infection (24).

CMV is a common β-herpesvirus with known lifelong latency following primary infection, which is typical for all herpesviruses (25). The reported seroprevalence rate in healthy adults ranges from 40% to 100% with ethnical differences (26, 27). However, CMV is not a highly contagious virus. In childhood, most infections occur through urine and saliva transmission. Other routes of transmission are sexual or through transmission of blood products or during organ donation. CMV infection shows a very complex course, divided into three subtypes. Subtype 1 reflects a primary infection without a previous infection with CMV and thus a lack of immunity (28). Since there are no typical symptoms of primary CMV infections except for the well-known infectious mononucleosis, the infection usually runs unnoticed due to the strong T cell response preventing uncontrolled viral replication in the host during an infection (29). After the first infection with the virus, a latency period begins allowing reactivation of the virus at any time (28). However, a weakened cellular immune response leads to reactivation of CMV and an increased viral load referred to as subtype 2, contributing to organ damage including heart involvement with pericarditis as in the case presented here. If an individual with a previous infection and an immune response becomes infected again with an CMV, e.g. in the case of a contaminated blood transfusion, this is referred to as a subtype 3 (28). Furthermore, CMV is the most common opportunistic infection in individuals with HIV, after hematopoietic stem cell or solid organ transplantation. Immunity against CMV controls its replication, although intermittent viral shedding can occur in seropositive immunocompetent cases (30). Reactivation of CMV is a failure of the T cell compartment to maintain control of its infection and is supposed to occur more frequently with increasing age due to adaptive immunosenescence (31). Moreover, vaccination is a strong stimulator of the immune system, coordinating a humoral and cellular adaptive immunity to polarize a vaccine-induced T cell response, also reported for SARS-CoV-2 vaccines (32). Vaccine-induced reactivation of CMV may have similarities with immune reconstitution inflammatory syndrome (IRIS) characterized by a paradoxical worsening of preexisting infection due to a reconstituted capacity for an inflammatory response following the initiation of antiretroviral therapy (ART), also reported in COVID-19 (33, 34). In addition, CMV reactivation is known to trigger an inflammatory response often persisting in COVID-19 patients long after SARS-CoV-2 is no longer detectable, which might be relevant for patients with long COVID that still have symptoms months after SARS-CoV-2 infection (35, 36). We are aware that this is the first report of an association between SARS-CoV-2 vaccination and CMV reactivation during ongoing vaccination programs worldwide, but clinicians should be sensitized for this complication and encouraged for appropriate diagnostic testing.

## Conclusions

Worldwide, huge vaccination programs are ongoing and post-marketing surveillance systems with assessment of vaccine safety are important for the detection of any events associated with SARS-CoV-2 vaccination. In view of the hundreds of millions of individuals to be vaccinated against SARS-CoV-2, a potential causal association with CMV reactivation may result in a large number of cases with potentially severe complications. With this case report, we aim to sensitize clinicians for viral disease as well as reactivation after vaccination and encourage appropriate diagnostic testing. Fortunately, treatment of CMV infection is possible and yielded a very good outcome for our patient. Further studies must provide insights into CMV reactivation after SARS-CoV-2 vaccination and possible recommendation of routine testing.

## Data Availability Statement

The original contributions presented in the study are included in the article/supplementary material. Further inquiries can be directed to the corresponding author.

## Ethics Statement

Ethical review and approval were not required for the study on human participants in accordance with the local legislation and institutional requirements. The patients/participants provided their written informed consent to participate in this study. Written informed consent to publish this report was obtained directly from the patient.

## Author Contributions

BT conceived the case report, collected and analyzed data, and wrote the manuscript. MP, KM, DT, and BT were directly involved in the treatment of the patient. JK and AS evaluated cardiac MRI findings and provided images. All authors contributed to the article and approved the final version.

## Funding

We acknowledge support from the Open Access Publication Funds of the Georg August University Göttingen.

## Conflict of Interest

The authors declare that the research was conducted in the absence of any commercial or financial relationships that could be construed as a potential conflict of interest.

## Publisher’s Note

All claims expressed in this article are solely those of the authors and do not necessarily represent those of their affiliated organizations, or those of the publisher, the editors and the reviewers. Any product that may be evaluated in this article, or claim that may be made by its manufacturer, is not guaranteed or endorsed by the publisher.
